# Quantifying intracellular *Mycobacterium tuberculosis*: An essential issue for in vitro assays

**DOI:** 10.1002/mbo3.588

**Published:** 2018-02-27

**Authors:** Deisy Carolina Rodriguez, Marisol Ocampo, Luz Mary Salazar, Manuel Alfonso Patarroyo

**Affiliations:** ^1^ Universidad Nacional de Colombia Bogotá Colombia; ^2^ Fundación Instituto de Inmunología de Colombia (FIDIC) Bogotá Colombia; ^3^ Universidad del Rosario Bogotá Colombia

**Keywords:** bacterial quantification, cytometry, fluorescence, infection assay, tuberculosis

## Abstract

Many studies about intracellular microorganisms which are important regarding diseases affecting public health have been focused on the recognition of host–pathogen interactions, thereby ascertaining the mechanisms by which the pathogen invades a cell and makes it become its host. Such knowledge enables understanding the immunological response triggered by these interactions for obtaining useful information for developing vaccines and drugs. Quantitative cell infection assay protocols are indispensable regarding studies involving *Mycobacterium tuberculosis*, which takes the lives of more than 2 million people worldwide every year; however, sometimes these are limited by the pathogen's slow growth. Concerning such limitation, a detailed review is presented here regarding the different methods for quantifying and differentiating an intracellular pathogen, the importance of mycobacteria aggregate dissociation and multiplicity of infection (MOI) in infection assays. The methods’ differences, advantages, and disadvantages are discussed regarding intra and extracellular bacteria (on cell surface) differentiation, current problems are outlined, as are the solutions provided using fluorophores and projections made concerning quantitative infection assays.

## 1 INTRODUCTION

Intracellular pathogens such as *Mycobacterium tuberculosis* (*M. tuberculosis*) (the main cause of tuberculosis) and parasites such as *Plasmodium falciparum* (the main cause of malaria), added to the human immunodeficiency virus (HIV) causing acquired immunodeficiency syndrome (AIDS) are recognized by the World Health Organisation as being responsible for a third of annual deaths in developing countries (WHO, 2017).

Many studies regarding these pathogens have been orientated toward recognizing host–pathogen interactions (Silva, 2012) thereby making in vitro cell infection assays indispensable. Recognizing and quantifying a pathogen in the intracellular or extracellular space (on the surface) has played a significant role and has inspired researchers to develop new methodologies thereby facilitating more rapid and effective detection of diseases. Developing differential staining techniques, using antibody fluorophore conjugation, producing modified mediums accelerating growth or facilitating bacteria quantification in less time, flow cytometry and the fluorometry represent some of the results obtained; most of them have been used regarding *M. tuberculosis* studies, pathogen with which this review is concerned.

Quantitative intracellular pathogen techniques have been widely used in developing new drugs and vaccines, studying intracellular pathogens’ interactions with their hosts and basic research associated with many pathogens’ intracellular development.

The current work has highlighted seven quantification methodologies used in *M. tuberculosis* studies: colony‐forming unit (CFU) on agar plates, microscope staining of acid‐alcohol‐resistant bacteria, fluorescent labeled antibodies against intracellular bacteria, FITC‐biotin‐avidin (FBA) staining of intracellular bacteria, RT‐PCR quantification of viable *M. tuberculosis*, flow cytometric quantification of intracellular bacteria and fluorometric methods of intracellular pathogens; each method's advantages and disadvantages are discussed here.

## 2 INFECTING PHAGOCYTIC AND NONPHAGOCYTIC CELLS

Pathogens can enter different types of cells by phagocytic and nonphagocytic pathways; the most studied ones regarding evaluation of intracellular pathogens have been related to phagocytosis described by Gordon, 2016. Phagocytosis now is known as a mechanism regarding host response to injury and infection. It has been defined as the process where pathogens or scavengers greater than 0.5 μm in size are recognized by phagocytic cells through specific pathogen‐recognition receptors (Stossel, 1999). A phagosome having engulfed microbes and scavengers then becomes formed, normally in macrophages; it becomes fused to a lysosome to form a mature phagosome where bacteria is degraded due to lysozymes and strong oxidant agents (Awuh & Flo, 2017; Richards & Endres, 2014).

Macrophages are the main phagocytic cells and are indispensable mediators of inflammatory processes (Duque & Descoteaux, 2014), playing an important role in innate and adaptive immune responses. Their precursors, monocytes, can be differentiated into classically activated proinflammatory (M1) or alternatively activated (M2) types depending upon cytokine microenvironment; both have plastic, rapid, and fully reversible activation (Mulder, Banete, & Basta, 2014; Porcheray et al., 2005).

*Mycobacterium tuberculosis* is one of the most successful pathogens; it has evolved to avoid phagolysosome maturation and can live in macrophages. Its evasion mechanisms have been widely studied finding specific facts such as; *M. tuberculosis* masks pathogen‐associated molecular patterns using phthiocerol dimycoceroserate (PDIM) lipids and can recruit MyD88‐independent macrophages, producing less inducible oxide synthase (iNOs) compared to independent macrophages (Cambier et al., 2014). It has also been demonstrated that *M. tuberculosis* induces macrophages type I activation (classically activated) and uses phagocyte lipids as a nutritional source (Benoit, Desnues, & Mege, 2008; McClean & Tobin, 2016).

*Mycobacterium tuberculosis* has as target cells phagocytic cells, particularly alveolar macrophages and lung dendritic cells, which constitute the majority of lung antigen presenting cells and defense against pulmonary infection. Phagocytic human (U937 monocyte cell line derived‐macrophages and THP‐1) and murine macrophage cell lines (RAW 264.7 and J774.2) have thus been used as in vitro cell model; the U937 cell line has also been used when evaluating the genes required for *M. tuberculosis* intracellular survival (Wei et al., 2000). Recently published studies (having different objectives) have involved macrophages and used essentially the same technique for quantifying intracellular mycobacteria: colony‐forming units (CFU) count and *Mycobacterium smegmatis* as model. One such was aimed at evaluating the role of macrophage membrane cholesterol (THP‐1 cell line) regarding mycobacterial entry to such host cells (Viswanathan, Jafurulla, Kumar, Raghunand, & Chattopadhyay, 2015). *Mycobacterium smegmatis* was used as model and methyl‐β‐cyclodextrin for host cell membrane cholesterol depletion; CFU count following infection revealed a significant reduction in mycobacterial entry to the macrophage, thereby highlighting the need for an optimum amount of cholesterol in plasmatic membrane for mycobacterial phagocytosis.

Hundreds of publications have shown the importance of studying phagocytosis and thus quantifying intracellular pathogens, a topic ranging beyond cells not normally considered phagocytic. An example would be a recent study demonstrating that B‐cell lymphoma line (Raji) cells have phagocytic processes for internalizing viable or dead *M. tuberculosis* where opsonization is crucial and that mycobacteria's intracellular presence triggers immune response factors such as immunoglobulin M (IgM) and costimulatory molecules (CD80 and CD86) production (Zhu et al., 2016).

It has also been demonstrated that *Mycobacterium* species can enter different cells (e.g., macrophages, epithelial cells, and neurons). *Mycobacterium smegmatis* (which is not pathogenic) enters and increases its virulence when passing through A549 adenocarcinomic human alveolar basal epithelial cells, although becoming degraded over a certain period of time (García‐Pérez, Hernández‐González, García‐Nieto, & Luna‐Herrera, 2008; Kim et al., 2011). *Mycobacterium leprae* infects epithelial, muscle, and Schwann cells (neurolemocytes) (Schorey et al., 1995) and *M. tuberculosis* and other species from the *M. tuberculosis* complex (MTC) enter epithelial cells and neurons to continue replicating within them (Bermudez & Goodman, 1996; Bermudez, Shelton, & Young, 1995; Shepard, 1955a).

The CFU method is usually used in detecting infection caused by bacteria or mycobacteria in nonphagocytic cells for studies of phagocytosis and sometimes for verification by different microscope techniques. This underlines the fact that quantifying intracellular bacteria is indispensable for studying infectious process regarding both phagocytic processes and nonphagocytic systems.

## 3 MYCOBACTERIUM DISSOCIATION AND MULTIPLICITY OF INFECTION‐BASED SELECTION REGARDING INFECTION ASSAYS

An *M. tuberculosis* virulence factor which is characteristic of mycobacteria is bacillus aggregate formation in winding‐curved cord attributed to the presence of mycoside trehalose‐6, 6′‐dimycolate. Increasingly larger aggregations are made in culture mediums depending on culture time; this is a source of difficulties since it has been seen to hamper entry to target cells in in vitro studies; the bacteria must thus be as dissociated as possible when infecting target cells.

It must be considered that mycobacteria are usually cultured in Middlebrook 7H9 liquid medium or Middlebrook 7H10 solid growth medium supplemented with oleic albumin dextrose catalase (OADC) and that growth in solid medium makes colony disaggregation more difficult. Different ways of disaggregating mycobacteria have been found by: adding glass pearls followed by constant shaking, which has also been used for dissociating colonies from solid mediums (Kahn & Schwarzkopf, 1933); Tween 80 can be added to liquid culture medium according to the manufacturer's indications, helped by constant shaking during culture time, this is sometimes changed for Tyloxapol (a nonhydrolyzable detergent), as happens in metabolomics studies (de Carvalho et al., 2010). Another method involves passing a mycobacterial pellet suspension through very small caliber needles five or six times; 26 ½ (Tiwari, Kannan, Vemu, & Raghunand, 2012; Virji, Makepeace, Ferguson, Achtman, & Moxon, 1993) and 26 and 25 caliber tuberculin syringes have been used (Sattler, Monroy, & Soldati, 2013; Schaible, Sturgill‐Koszycki, Schlesinger, & Russell, 1998), passage occasionally following infection to give a uniform CFU count. Also was found the rapid stirring on ice in a overhead stirrer to avoid agglutination, followed by passage through a 0.8 μm membrane to guarantee a predominance of bacteria just in controls by Ziehl–Neelsen staining (Shin, Han, Manning, & Collins, 2007). Lastly, there is also gentle sonication over short periods of time, for example, at 30 W for 5 min (Chapeton‐Montes, Plaza, Barrero, & Patarroyo, 2007; Kim et al., 2011).

**Figure 1:**
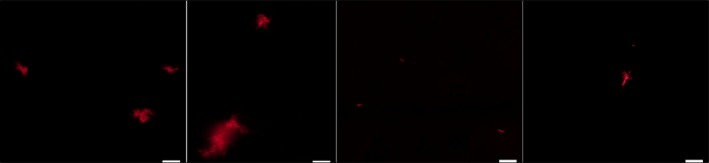
*Mycobacterium tuberculosis* H37Rv dissociation using tuberculin syringe with cut needle and rhodamine B staining. The first two photographs show characteristic groups without dissociation. White bar size is 100 μm

Passage through a syringe has been found to be the cheapest and most accessible form of disaggregation by our research group; it has led to excellent results and its use has been referred to more frequently in recent publications. Nonremovable (i.e., fixed needle) tuberculin syringes which needle has been broken using sterile forceps prior to use with mycobacteria, can be used to avoid the severe risk of puncture (regarding the disadvantage of using needles in biosafety level 3 laboratories). Even after having cultures in Tween medium with aggregations containing a maximum of 15 bacilli (few) under microscope examination with Ziehl–Neelsen staining. Figure 1 compares mycobacteria before being disaggregated and after being disaggregated with tuberculin syringe following five passes and stained with rhodamine B; aggregate reduction is evident as is the presence of independent bacilli. Very few aggregates contain more than five bacilli; such results coincided with some obtained for *Mycobacterium marinum* (Takaki, Davis, Winglee, & Ramakrishnan, 2013), the authors stating that they had just independent bacilli by making an additional passage through 5 μm membranes, thereby losing much mycobacterial mass.

In addition to having mycobacteria dissociated as much as possible, there must also be optimum multiplicity of infection (MOI) to be real at the moment of infection, thereby ensuring target cell viability. Several MOI have been reported but a 1:10 MOI (10 bacteria per target cell) is usually found, confirming cell viability is maintained after periods of infection (Wei et al., 2000). MOI lower than 1: 1 and 1:5 have been used for quantifying CFU (Sethi et al., 2016) and higher ones, such as 1:50; the maximum MOI reported to date has been 1:100, referred to in some articles as associated with preparing samples for microscopy. There have been no reports of MOI ‐associated cell death (Geijtenbeek et al., 2003; Kim et al., 2011). MOI are usually confirmed by sowing in agar dishes and CFU count by optical density after mycobacterial dissociation.

## 4 QUANTIFYING INTRACELLULAR BACTERIA

### 4.1 Technique 1: CFU‐based intracellular bacteria count

The first report of intracellular *M. tuberculosis* in epithelial cells was published in 1955 (Shepard, 1955b), that is, infecting Hela cells (the first cell line to be cultured in the laboratory). This supported a later study for evaluating a *Yersinia pseudotuberculosis* plasmid inserted into *Escherichia coli* K12 enabling bacteria to invade Hep‐2 epitelial cells. This study described the CFU count technique in detail; 8 × 10^5^ cells were incubated in 24‐well culture plates thereby enabling their adherence, followed by infecting them with 6 × 10^7^ transfected bacteria for 3 hr at 36°C. Extracellular bacteria were eliminated by 10 washes with sterile PBS and treatment with gentamicin (an aminoglucoside which was later understood to be able to enter mamal cells, though it was questioned whether it could be very effective against modified bacteria). Following antibiotic treatment, the cells were lysed with 1% Triton X‐100 in deionized water and plated on agar. The experiment was replicated by substituting cell lysis for fixation with 2% glutaraldehyde and preparation for visualization by electron microscope (Isberg & Falkow, 1985). This procedure can be considered as a “final methodology” since it has not undergone any important variations up to today.

Some researchers have changed 1× PBS for Hank's balanced salt solution (HBSS) or growth medium and varied the number of washes; they have also varied the solutions used for cell lysis, 100× Triton, SDS (Zhu et al., 2016), 0,1% saponin solution (McKinney et al., 2000) and/or distilled or deionized water being frequently used (Wei et al., 2000). The antibiotics used vary according to the pathogen being studied; for example, a 20 μg/ml amikacin concentration is usually used for *M. tuberculosis* in incubation periods of 2 hr, some washes have been made and intracellular mycobacteria plated on 7H10 culture medium supplemented with OADC (Bermudez & Goodman, 1996; Bermudez & Young, 1994).

*Mycobacterium tuberculosis* colony count can be obtained from cell lysis after 20 days’ culture; this is a great disadvantage in studies involving this pathogen. Using lecithin in culture mediums, using blood sugar agar mediums instead of Middlebrook and adding ascorbic acid as antioxidant (or other supplements) have been proposed for accelerating its growth and enabling colonies to be seen in less time. It has been reported that using a microaerophilic atmosphere and binocular microscope have led to reducing the time in which colonies can be appreciated in just 1 week's culture, even when starting from tuberculosis patients’ sputum (Ghodbane, Raoult, & Drancourt, 2014) being very useful for *M. tuberculosis* in vitro and diagnostic assays.

A great disadvantage of the CFU count method is that most antibiotics rely on different mechanisms for entering eukaryotic cells; thus, stating that the mycobacteria being attacked are just those in the extracellular space is not entirely true since. It has been confirmed that amikacin, the antibiotic most used in *M. tuberculosis* infection assays, does enter dendritic cells (Montes‐Worboys et al., 2010). Some publications have stated that only washes have been done to remove mycobacteria from the extracellular space, having cell envelope‐associated mycobacteria as positive proof of infection and thus adding error to the procedures. It cannot be determined whether a target cell can internalize mycobacteria or any type of dead bacteria with this method, nor study the process required for entry and/or any inherent implications.

It was considered nonappropriate the CFU counting method in studies of intracellular quantification of mycobacteria requiring evaluate intracellular growth since its natural growth involving aggregate formation of mycobacterias. The amount of colonies a researcher might count may not represent the real amount of mycobacteria in one assay, unless some effective mycobacteria dissociation method is used following the intracellular growth period.

In spite of this method's numerous disadvantages, it is the most reliable and most used worldwide, probably due to it being practical when making a count, the colonies which can be seen are viable mycobacteria and it can be determined whether an antibiotic treatment is having an effect due to an appreciable reduction in the amount of colonies compared to untreated control.

### 4.2 Technique 2: Staining for microscope detection of acid‐alcohol‐resistant bacteria

Even though staining techniques for detecting acid‐alcohol bacteria were proposed as a diagnostic method in 1882, it took a long time until they were used for intra and extracellular mycobacteria detection, quantification or even differentiation. The methodology proposed by Strahl, Gillaspy, & Falkinham (2001) involved infecting protozoa from the species *Tetrahymena pyriformis* with different mycobacteria strains for evaluating phagocytosis and intracellular growth. Smears were prepared on glass slides and stained with Ziehl–Neelsen and O‐rhodamine B following standard procedures; the slides were observed by Zeiss Axiophot microscope (545 nm *L*_ex_ and 610 nm *L*_em_). The authors argued that changing the focal plane when visualizing protozoa after rhodamine staining enabled differentiating bacteria within them which were bound outside a cell. They analyzed 50–100 protozoa without observing extracellular aggregates having more than 15 mycobacteria.

Ziehl–Neelsen and auramine‐O detection techniques have been modified for improving *M. tuberculosis* detection in cerebrospinal fluid from patients’ samples (Chen et al., 2012). The technique involved fixing samples with 4% p‐formaldehyde instead of using heat‐fixed slides and then (after drying them) permeabilizing them with 0.3% TritonX‐100 for 30 min. Ziehl–Neelsen staining followed conventional procedure and the manufacturer's recommendations, but acid‐alcohol in Triton solution was used and methylene blue acting for 5 min. Microscope observation revealed mycobacteria in 100% of patients’ samples compared to 27.6% detection sensitivity for standard staining. Auramine‐O permeabilized samples were stained according to manufacturer's recommendations, followed by fluorescent immune‐labeling for delimiting the membrane, using different biotin‐binding antibodies providing fluorescent labeling with a streptavidin conjugate: anti‐CD11b, anti‐ED1, anti‐CD3, and anti‐CD20. Fluorescent labeling confirmed that the modified Ziehl–Neelsen technique enabled high sensitivity intracellular mycobacteria staining, differentiation and quantification using a conventional optical microscope. However, this technique has disadvantages regarding microscope observation for intracellular count, the fact that a very small amount of cells can be analyzed and human error when deciding whether mycobacteria is within cells, on their surface or outside them, very valuable information regarding studies involving invasion assays.

### 4.3 Technique 3: Quantifying intracellular bacteria (including fluorescent antibody labeling)

Different methodologies have been developed to accelerate the intracellular quantification of pathogens, taking advantage of fluorophores in terms of sensitivity and the options currently offered by detection and analysis equipment, each method has advantages and disadvantages. Almost all the studies mentioned used the CFU count method for comparison purposes, standardizing methodologies and confirming results.

Quantifying intracellular bacteria which includes fluorescent labeling with antibodies is the first on the list and was designed in 1985 (Heesemann & Laufs, 1985). In spite of having some modifications according to the pathogen, the cells being studied and the introduction of new fluorophores, the procedure's principles remain the same. After the cells had been infected, they washed with saline buffer to remove excess extracellular bacteria, followed by selective labeling with antibodies. An extracellular bacteria‐recognizing primary antibody is added (since these cannot enter cells); unbound antibody is then removed by washes. A fluorophore‐labeled secondary antibody, usually tetramethylrhodamine isothiocyanate (TRITC) that emits red light, recognizes bound antibodies to the bacteria. Following incubation, the unbound secondary antibody was removed by washing; the cell membrane was then permeabilized with methanol or a detergent solution and labeled with antibodies as described for extracellular bacteria, but this time a secondary antibody is used which had been labeled with another fluorophore emitted in a different region of the spectrum (fluorescein isothiocyanate (FITC)‐green).

Most extracellular bacteria were then stained with TRITC, whereas intracellular bacteria were only fluoro‐marked with FITC. The bacteria were visually quantified by fluorescence microscope. This technique has similar previously mentioned disadvantages regarding the use of microscopy; in this case accompanied by a great increase in assay time using antibodies and the amount of washes needed for fluorescent staining to be reliable. However, the time to obtain the results is shorter compared to the method of colony count.

### 4.4 Technique 4: Quantifying intracellular bacteria by FBA staining

The acronym FBA refers to using FITC, biotin and avidin for staining and detecting intracellular bacteria following Agerer's methodology (Agerer, Waeckerle, & Hauck, 2004). The assay involved using transfected 293T cells (mouse fibroblasts) which are adherent cells and became infected by different previously fluoro‐marked bacteria (*Neisseria gonorrhoeae* NS11‐B2.1 and NS11‐B2.1, *Staphylococcus carbonosus*,* S. aureus*).

Labeling was done by suspending the bacteria in a 0.4 mg/ml FITC solution in phosphate buffer (PBS) and incubating for 15 min at 4°C; the same amount of 0.3 mg/ml biotin solution was added and left for a further 30 min. The solution was washed and biotinylated FITC‐labeled bacteria were obtained; 40 bacteria per cell were infected and incubated for two hours, after which the samples were fixed with 4% paraformaldehyde in PBS, washed thrice and incubated with 5% blocking solution (10% bovine fetal serum [BFS] in PBS). The extracellular bacteria were then stained with streptavidin Alexa‐647 (which reacted with the biotin). Following permeabilization for 5 min with permeabilization buffer (PBS with 10% BFS and 0.2% saponin), they were stained with Alexa Fluor‐546 for 30 min for visualizing cell cytoskeleton. The samples were observed by LSM 510 confocal scanning laser microscope. The authors stated that there had been an appreciable reduction in time regarding assays with antibodies and better differentiation between intra‐ and extracellular bacteria due to cell cytoskeleton staining. It is certainly an advantageous technique over others using a microscope; however, it has the disadvantage of being limited by microscope count.

### 4.5 Technique 5: Quantifying viable *Mycobacterium tuberculosis* by RT‐PCR

A bacterial detection and quantification techniques which has been used during recent decades (especially in the area of diagnosis) involves using quantitative polymerase chain reaction (qPCR). Two RT‐PCR diagnostic techniques were developed in 2003 for *M. tuberculosis* detection. This technique involves the N‐acetyl‐l‐cysteine‐NaOH procedure for purifying and homogenizing patients’ samples; they are then centrifuged and the pellet is plated in a BACTEC system and Ziehl–Neelsen stained as control. The remainder is used for DNA extraction, a synthetic calibrator being added prior to DNA extraction for controlling procedure efficiency. The primers were designed from two different regions of the *M. tuberculosis* genome; the first consisted of an IS6110 multicopy insertion element which has been found in all clinical *M. tuberculosis* isolates, whereas the second was part of a two‐component regulatory system (2CRS) senX3‐regX3 IR, having mycobacterial interspersed repetitive units (MIRUs), which has only been described in MTC strains. UV spectrometry was used for quantifying reference curves and (since senX3‐regX3 IR was designed for amplifying a single sequence for each *M. tuberculosis* genome) bacterial load was directly proportional to DNA load measured by this technique (Broccolo et al., 2003). It is not limited to diagnostic use; suitable selection of genetic material enables quantifying mycobacteria having specific characteristics and it can be applied in in vitro and in vivo tests.

This technique can be applied in vivo for *M. tuberculosis* replication follow‐up in infected mice. It has been proposed that plasmid pBPIO is a circulating plasmid carrying a kanamycin resistance marker, coordinated with bacterial DNA, becoming lost at constant and quantifiable speed in cell division. A Biorad iCycler was used for RT‐PCR with iQ SYBR Green Supermix. The quantification curve was constructed with genomic *M. tuberculosis* DNA. CFU quantification in selective and nonselective medium led to determining division rates regarding *M. tuberculosis* infection progress (practically zero in chronic tuberculosis and hypoxic states). This method can be used for determining a system's latent bacterial load, using just RT‐PCR, which can be very useful in both in vivo and in vitro systems (Gill et al., 2009).

Including markers such as propidium monoazide (PMA) or ethidium monoazide bromide (EMA) in PCR techniques enables differentiating and quantifying viable bacteria. Its standardization for use in *M. tuberculosis* has been proposed for quantifying viable mycobacteria in patients’ samples as a diagnostic method. Sputum samples were treated with 50 mg/ml 25% N‐acetyl‐l‐cysteine since *M. tuberculosis*‐induced cell death has occurred at higher concentrations. Both aforementioned markers were evaluated in this approach; however, EMA was found to inhibit viable cell amplification, PMA thus being preferred. The exposure conditions for the genetic material obtained from the samples for optimal quantification was 100 μmol/L PMA for 5 min and 1 min exposure at 650w for halogen lamp analysis. The amplification primer contains an inhA‐mabA intergenic region having no homology with other pathogens or eukaryotic cells.

A DNA concentration curve with complementary ethidium bromide staining enables ascertaining the exact amount of bacilli in each sample, differentiating viable from nonviable ones. This method had greater sensitivity than Ziehl–Neelsen staining and excellent correlation with sputum culture (de Assunção et al., 2014). Although there are still no reports regarding this technique's use in in vitro tests and it does not enable differentiating intracellular bacteria, it was been extremely useful in high accuracy quantification of bacterial load (even in small samples) and together with a good selection of stains it can be used in multivariate analysis of bacterial replication, viability and infection. This technique's drawbacks concern are the time taken for obtaining plasmids, the high cost of stains, extraction reagents, and specialized RT‐PCR equipment.

### 4.6 Technique 5: Quantifying intracellular bacteria by flow cytometry

Pils, Schmitter, Neske, and Hauck (2006) followed the previously described FBA staining protocol; however, they proposed a parallel protocol which included flow cytometry for quantifying *N. gonorrhoeae* (Pils et al., 2006). Some modifications were necessary for enabling bacteria count by cytometer. Bacteria were only labeled with carboxyfluorescein succinimidyl ester (CFSE) and then incubated with cells (20:1) for two hours. Infected cells were detached from the dish by trypsin treatment and washed twice with 1% PBS. Infected cells were suspended in 1 ml flow buffer (1% BFS in PBS). Intracellular bacteria were discriminated from extracellular bacteria by adding trypan blue (0.2% final concentration) immediately before reading to deactivate extracellular bacteria fluorescence. It has been suggested that trypan blue were unable to enter viable cells containing bacteria within them but they could surround the bacteria on their exterior, absorbing the light emitted by them; the equipment could only receive a signal from intracellular bacteria. In the study, 10,000 cells per sample were read using a FACSCalibur cytometer (Becton Dickinson/FL1 detector); CFSE had green emission collected in the FACScan's FL1 detector; the technique has been successfully modified and evaluated for *M. tuberculosis* count (Chapeton‐Montes et al., 2007; Ocampo, Curtidor, Vanegas, Patarroyo, & Patarroyo, 2014; Ocampo et al., 2012).

Cytometry has more advantages than other techniques regarding analyzing large‐sized samples, making them statistically representative, and it is even more advantageous as it can enable intra and extracellular bacteria differentiation. However, a flow cytometer increases processing costs, especially considering that not all laboratories having cytometers can process pathogens such as *M. tuberculosis*.

The discovery of the green fluorescent protein (GFP) has revolutionized studying host–pathogen interactions (Dhandayuthapani et al., 1995; Valdivia, Hromockyj, Monack, Ramakrishnan, & Falkow, 1996), and thus cellular infection assays, it has replaced some fluorophores providing a variety of advantages. For example, *Mycobacterium smegmatis* has been used, after having been transformed by pMSP12::DsRed2 plasmid electroporation (Cosma, Humbert, & Ramakrishnan, 2004; Tiwari, Soory, & Raghunand, 2014; Tiwari et al., 2012; Viswanathan et al., 2015), for studying the cell membrane regarding phagocytic processes, or determining *M. tuberculosis* H37Rv virulence factors in in vitro (Hanekom et al., 2003) and in vivo studies with *Mycobacterium marinum*,* Staphylococcus aureus,* and *Pseudomonas aeruginosa* constitutively expressing an GFP, Wasabi or tdTomato fluorescent proteins (Cambier et al., 2014).

Fluorescent protein expression vectors have also facilitated evaluating gene regulation within a host cell in cell invasion assays; for example, a study using the pLL192 plasmid (Srivastava et al., 2007) involved designing promoter vectors for expressing different *M. tuberculosis* proteins combined with GFP reporter (*hsp60*) and kanamycin resistance (*kan*) genes obtained in *E. coli* and electroporated in *Mycobacterium bovis BCG* and *M. tuberculosis*. Mouse alveolar macrophages (J744 cell line) infected at 1:4 MOI with transfected bacteria were washed and treated with kanamycin to kill remaining extracellular bacteria or those bound to the surface. Cells were lysed with an SDS solution and an aliquot was sown in Middlebrook 7H10 medium with antibiotics for CFU count. Analyzing bacteria by flow cytometry revealed greater fluorescence in those overexpressing proteins when submitted to stress within macrophages, considering them to be genes having an adaptive response to mycobacteria (*mceA1* is one of them). Other genes identified by GFP protein expression have also been evaluated regarding infection of different cell lines and some have been determined to be virulence factors (Velmurugan et al., 2007; Zahrt & Deretic, 2000, 2001).

### 4.7 Technique 7: Fluorometric method for quantifying intracellular pathogens

A new cytometry‐based β‐lactamase fluorometric method for quantifying *Neisseria gonorrhoeae* infected cells and differentiating intracellular from extracellular bacteria (Bish, Song, & Stein, 2008) has been successfully designed for tracking intracellular *M. tuberculosis* vacuole rupture (Keller et al., 2013). THP‐1 macrophages were platting 5 × 10^5^ cells/well and incubated with 30 nmol/L Phorbol Myristate Acetate 2 days before infection.

Infection involved β‐lactamase‐expressing mycobacteria being dissociated by sonication, filtered through a syringe and put onto cells at 1:1 MOI. Infected cells were loaded with CCF4‐AM (a β‐lactamase‐sensitive FRET reporter) reading at 535 nm (green). β‐lactamase on mycobacterial surface was able to cleave CCF4 substrate as soon as phagosome rupture occurred, turning cells blue (reading at 450 nm), being uninfected ones green. Cells did not change in an assay with *Mycobacterium bovis* BCG (a nonvirulent strain) because it resides in phagosome compartment. Samples should be analyzed by epifluorescence or confocal microscope enabling automated fluorescence signal scoring for each cell.

This is a very expensive method due to the high cost of CCF4‐AM substrate and analysis equipment, although it is advantageous regarding real‐time analysis and knowledge of host–pathogen interaction in terms of quantitative analysis. It does not provide information regarding the amount of mycobacteria which infected each cell; it can only show how many cells were infected and has the disadvantages involved in using microscopy (even though such new multiwell plate analysis systems allow rapid counting of hundreds of replicas). Fluorescent mycobacteria can be used for quantitative analysis ascertaining the amount of intracellular bacteria; they are emitted at different wavelengths to those mentioned beforehand and can be analyzed by flow cytometry (Simeone et al., 2015). Simeone's group has evaluated the role of *M. tuberculosis* in phagosome rupture in different types of phagocyte. This type of enzyme “ignition switch” is known as reporter enzyme fluorescence (REF) and has also been very useful in quantifying bacterial load in in vivo assays (usually by confocal microscopy) (Kong & Cirillo, 2010).

Studies currently including fluorometric analysis for quantifying bacteria have been done with bacteria and mycobacteria expressing different fluorescent proteins; for example, studies with zebra fish infected with *Mycobacterium marinum* have been very useful when evaluating macrophage recruitment and recognizing virulence factors. A protocol has been designed recently which has been based on fluorescence microscopy and fluorometry; it has been validated for evaluating pathogenesis and the efficacy of antimicrobial drugs after zebra fish have been infected (with *Mycobacterium marinum*) by microinjection (Takaki et al., 2013). A methodology has also been proposed for quantifying intracellular mycobacteria with *M. tuberculosis* expressing the green fluorescent protein for massive analysis by automated confocal microscopy (Queval et al., 2014).

## 5 THE PRESENT AND FUTURE OF TECHNIQUES FOR QUANTIFYING INTRACELLULAR PATHOGENS

**Figure 2:**
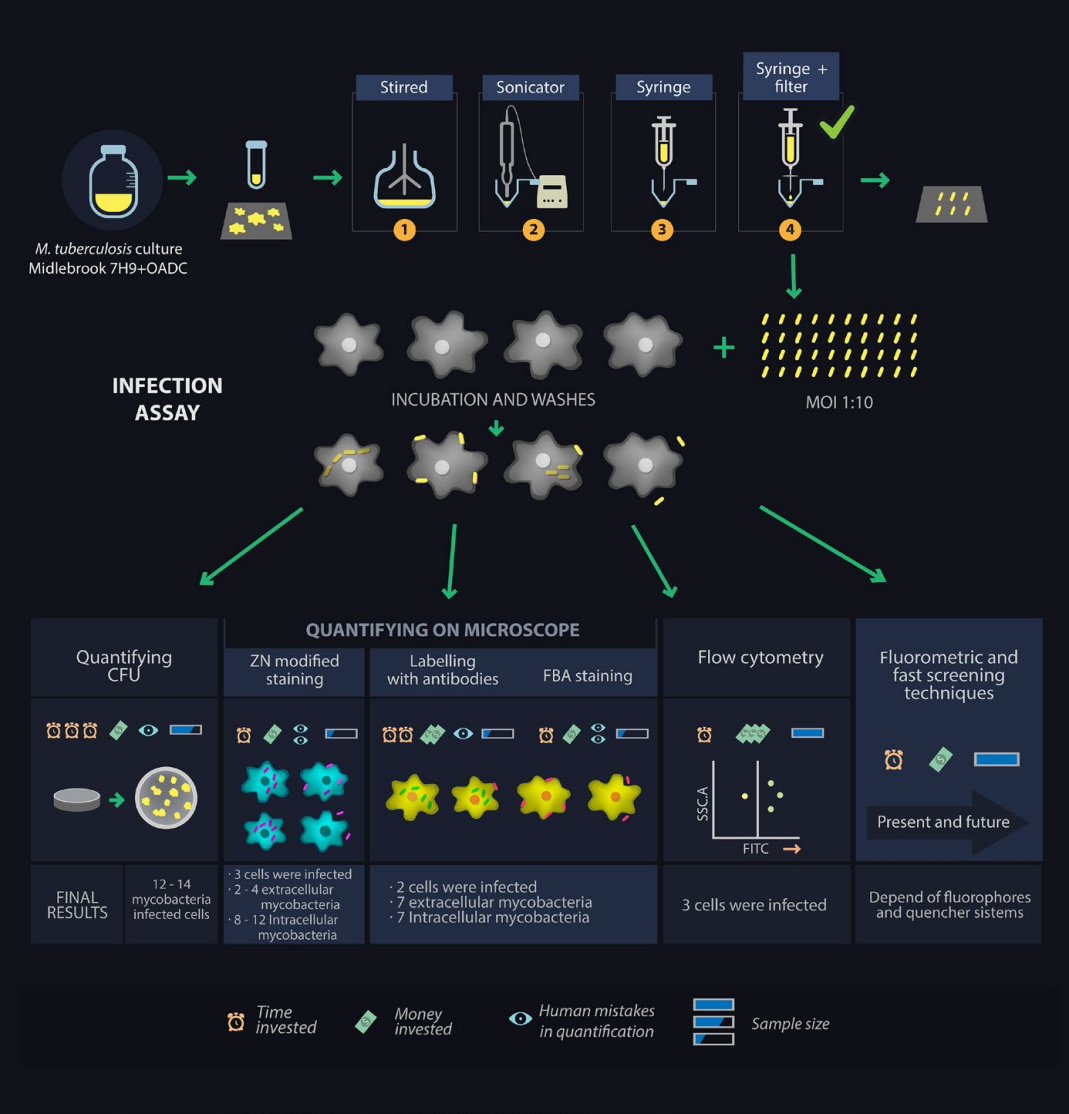
Overview of quantitative cell infection assay protocols, four ways of disaggregating mycobacteria are indicated with numbers

Advances made to date regarding the development of quantitative intracellular pathogen techniques, the trend toward fluorescence‐based analysis and each technique's limitations highlight the utmost importance of continuing to explore different techniques for quantifying intracellular microorganisms (Figure 2). These should be especially focus on *M. tuberculosis* quantification with methodologies enabling extracellular mycobacteria to be differentiated from those binding to target cell surface, overcoming the limitation of CFU count for an aggregate‐forming microorganism having prolonged growth time and avoiding the limitation regarding the significance of a sample being analyzed implicit in techniques involving the use of a fluorescence microscope. Developing rapid screening techniques based on measuring fluorescence, deactivation it or both are the present and future for the effective quantification and differentiation of intracellular pathogens; they are useful in developing drugs, vaccines and low cost diagnostic methods regarding both time and money.

## CONFLICT OF INTEREST

None declared.
